# Statins as Anti-Inflammatory Agents in Atherogenesis: Molecular Mechanisms and Lessons from the Recent Clinical Trials

**DOI:** 10.2174/138161212799504803

**Published:** 2012-04

**Authors:** Alexios S Antonopoulos, Marios Margaritis, Regent Lee, Keith Channon, Charalambos Antoniades

**Affiliations:** Department of Cardiovascular Medicine, University of Oxford, Oxford, United Kingdom

**Keywords:** Atherosclerosis, statins, inflammation, coronary heart disease, heart failure, outcome, endothelial nitric oxide synthase, vascular redox.

## Abstract

Ample evidence exists in support of the potent anti-inflammatory properties of statins. In cell studies and animal models statins exert beneficial cardiovascular effects. By inhibiting intracellular isoprenoids formation, statins suppress vascular and myocardial inflammation, favorably modulate vascular and myocardial redox state and improve nitric oxide bioavailability. Randomized clinical trials have demonstrated that further to their lipid lowering effects, statins are useful in the primary and secondary prevention of coronary heart disease (CHD) due to their anti-inflammatory potential. The landmark JUPITER trial suggested that in subjects without CHD, suppression of low-grade inflammation by statins improves clinical outcome. However, recent trials have failed to document any clinical benefit with statins in high risk groups, such in heart failure or chronic kidney disease patients. In this review, we aim to summarize the existing evidence on statins as an anti-inflammatory agent in atherogenesis. We describe the molecular mechanisms responsible for the anti-inflammatory effects of statins, as well as clinical data on the non lipid-lowering, anti-inflammatory effects of statins on cardiovascular outcomes. Lastly, the controversy of the recent large randomized clinical trials and the issue of statin withdrawal are also discussed.

## INTRODUCTION

Atherosclerosis has been widely recognised as an inflammatory process [1]. Statins, or 3-hydroxy-3-methylglutaryl coenzyme A (HMG-CoA) reductase inhibitors, are widely used in clinical practice as large randomized clinical trials have documented their benefits in primary [2, 3] and secondary [4, 5] prevention of cardiovascular disease (CVD). 

Evidence from both experimental and clinical studies supports the notion of “pleiotropic” effects of statins. In subjects with cardiovascular risk factors, statins reduce circulating C-reactive protein (CRP) and pro-inflammatory cytokines levels [6-8]. In human vessels, statins rapidly induce favorable effects on vascular redox state and reduce vascular reactive oxygen species (ROS) generation [9, 10]. In clinical studies statins consistently improve endothelial function in patients with or at risk for CVD [11-15]; they also reduce T-cell activation, macrophage infiltration, vascular wall inflammation, and promote plaque stabilization [16]. Statins induce additional vascular benefits when compared to other drug classes with similar low-density-lipoprotein (LDL) cholesterol lowering effects, such as ezetimibe [17, 18], while discontinuation of statins results in rebound inflammation and increased short-term cardiovascular risk, even in the absence of lipid levels fluctuation [19, 20]. Such observations suggest the pleiotropic effects by statins.

Further to lipid lowering, the anti-inflammatory effects of statins have been considered responsible for their protective effects in patients with coronary heart disease (CHD). The anti-inflammatory properties of statins are also likely to account for their role in primary and secondary prevention of stroke [21, 22], improvement of short-term outcome of acute coronary syndrome patients [23], reduction of the risk for atrial fibrillation post-coronary artery bypass grafting (CABG) [24] and in patients with heart failure [25] -all of which have a less clarified association with LDL lowering. The JUPITER (Justification for the Use of Statins in Prevention: an Intervention Trial Evaluating Rosuvastatin) study demonstrated that statin treatment reduces cardiovascular risk in primary prevention, even in healthy individuals with elevated CRP levels [26]. Such observations spurred initial pervasive enthusiasm by suggesting that statins could be used in several clinical conditions characterized by high inflammation. Nevertheless recent clinical trials in high-risk, heart failure and chronic kidney disease (CKD) patients have failed to document any benefit of statin treatment on clinical outcome [27, 28]. This review will specifically address the controversies related to recent large randomized clinical trials and possible indications for statin use in other disease states. 

## PLEIOTROPIC EFFECTS OF STATINS: THEIR IMPACT ON THE MEVALONATE PATHWAY 

Statins lower LDL cholesterol by inhibiting HMG-CoA reductase, the rate limiting enzyme in cholesterol biosynthesis. The putative, non-lipid related effects of statins are mediated by concomitant inhibition of protein isoprenylation, a process responsible for a variety of cellular responses downstream the mevalonate pathway. We have recently demonstrated that co-incubation of human vessels with statins and mevalonate reverses the beneficial effects of statins on vascular redox state [9, 10]; such findings highlight the importance of the mevalonate pathway in mediating the pleiotropic effects of statins in humans. L-mevalonate is a precursor in the formation of important isoprenoid intermediates such as farnesylpyrophosphate (FPP) and geranylgeranylpyrophosphate (GGPP). These molecules are responsible for post-translational modification of proteins by covalent addition of farnesyl or geranylgeranyl groups to cysteine residues [16]. This process is named “prenylation” and affects numerous signal transduction molecules in vascular and myocardial signaling pathways. An important group of such intracellular signaling pathways modulated by statins involves the small guanine triphosphate (GTP)-binding proteins, such as Rho, Rac and Ras [16]. These small GTP proteins regulate pro-atherogenic, pro-inflammatory pathways and are activated dependent of isoprenylation status. Rho proteins are involved in the expression of pro-inflammatory cytokines, as well as in the formation and maintenance of actin cytoskeleton. The Ras proteins regulate cell proliferation and hypertrophy, while Rac transduction signaling modulates ROS generation [29]. Further to modulation of small GTP-binding proteins activation, experimental evidence suggests direct activation of peroxisome proliferator-activated receptors (PPAR) -α and -γ by statins in platelets, inflammatory cells, vascular wall cells and cardiomyocytes [30, 31]. 

## STATINS AS ANTI-INFLAMMATORY AGENTS

An important study that addressed the anti-inflammatory effect of statins was the PRINCE (pravastatin inflammation/CRP evaluation) trial [32]. This trial demonstrated that oral administration of pravastatin (40mg/day) for 24 weeks significantly reduced serum CRP levels in subjects with and without CVD, independently of any changes in LDL cholesterol [32]. The only independent predictors of change in CRP levels were allocation to pravastatin and baseline CRP levels [32]. Although the PRINCE trial was not designed to assess clinical outcome endpoints, it confirmed the anti-inflammatory effects of pravastatin in the primary and secondary prevention setting. Other smaller clinical studies have demonstrated anti-inflammatory capacities to be a common drug class property of statins: they reduce CRP and/or circulating pro-inflammatory cytokines levels in patients with hypercholesterolemia [6], diabetes mellitus [8] and metabolic syndrome [7]. 

Further to statins, other hypolipidemic agents seem also to possess lipid-lowering independent effects. Although the pleiotropic properties of ezetimibe remain doubtful [17, 18], studies on fibrates and niacin have yielded important findings. Fibrates induce additive beneficial endothelial and vascular effects when added to statin monotherapy [33]. Niacin suppresses proinflammatory genes expression in vascular wall [34] and exerts beneficial effects on adipose tissue function. Adipose tissue has been lately recognized as a strong determinant of vascular homeostasis, via the release of adipokines [35]. Despite the possible anti-inflammatory effects of agents such as niacin and fibrates, statins lipid-lowering independent effects have been strongly associated with clinical outcome. Moreover statins are considered potent anti-inflammatory agents that have a wide range of anti-inflammatory effects in various tissues. 

### Endothelium-Specific Anti-Inflammatory Effects 

Nitric oxide (NO) is critical for maintaining endothelium homeostasis via its vasodilatory, anti-inflammatory and overall anti-atherogenic effects. It is widely accepted that statins favorably affect important pathways regulating NO bioavailability. They up-regulate endothelial nitric oxide synthase (eNOS) gene expression in human endothelial cells in an L-mevalonate- and GGPP-inhibitable way [36]. This effect is mediated by inhibition of Rho-kinases geranyl-geranyl-phosphorylation by statins [36], and results in increased Kruppel-like factor 2 expression - a strong regulator of eNOS expression [37]. Activation of PI3-Akt protein kinase pathway is an additional mechanism by which statins lead to increased eNOS gene expression and NO generation [29]. Moreover both lipophilic and hydrophilic statins induce polyadenylation of eNOS mRNA in a Rho-dependent way, by modulation of RNA polymerase II activity, a process that stabilizes eNOS mRNA [38]. Statins also down-regulate caveolin-1 expression in endothelial cells, a molecule which regulates eNOS subcellular localization and inactivates eNOS [39]. In addition, statins promote eNOS - heat shock protein 90 (Hsp90) interaction that phosphorylates eNOS at Ser1177 and enhances eNOS activation [40].

Further to these direct effects on eNOS gene expression and cytosolic abundance, statins favorably affect eNOS coupling (i.e. the enzymatic state where metabolism of L-arginine and shuttle of electrons is coupled with NO production instead of O_2_^-^). Asymmetrical dimethylarginine (ADMA) is now believed to be a key mediator of inflammation-induced endothelial dysfunction [41], as it induces eNOS uncoupling in advanced atherosclerosis [42]. Statins reduce circulating ADMA levels in patients with diabetes [43], hypercholesterolemia [44] or stroke [45]. *In vitro* studies have demonstrated that this effect is mediated by up-regulation of dimethylarginine dimethylaminohydrolase gene transcription by statins, the enzyme responsible for ADMA metabolism [46]. Moreover, statins increase the bioavailability of tetrahydrobiopterin (BH_4_), which is the critical eNOS co-factor that maintains the enzyme at its coupled form [47]. In cell studies on human umbilical vein endothelial cells (HUVECs), cerivastatin and fluvastatin increased GTP-cyclohydrolase I (GTPCH) mRNA expression, which is the rate limiting enzyme in *de novo* BH_4_ biosynthesis [48]. GTPCH up-regulation is a major mechanism accounting for statin-mediated increase in NO bioavailability in human vessels. We have shown that short-term treatment of coronary patients undergoing CABG operation with atorvastatin rapidly up-regulates vascular GTPCH expression and activity, resulting in improved vascular BH_4_ bioavailability, improved eNOS coupling and reduced vascular superoxide generation in the internal mammary arteries of these patients. These effects are mevalonate-inhibitable and dependent on reduction of Rac1 activation by atorvastatin [9]. 

In addition to improving NO bioavailability, endothelial dysfunction can also be restored by repair and regeneration of damaged endothelial cells. Simvastatin-induced vascular endothelial growth factor synthesis promotes endothelial healing in injured hamster arteries [49]. In heart failure patients rosuvastatin administration increases circulating endothelial progenitor cells and improves endothelial function [50]. 

Furthermore, statins inhibit endothelial cell activation, which is one of the first steps in atherogenesis. Inhibition of Rho-kinase by statins is responsible for attenuation of adhesion molecules expression in endothelial cells, such as intracellular adhesion molecule-1, independent of any effects on NO bioavailability [51]. In addition, von-Willebrand factor expression is down-regulated by statins via inhibition of the small GTPase protein cell division cycle 42 [52], while simvastatin and atorvastatin reduce matrix metalloproteinase (MMP)-9 expression and activity *in vitro* [53]. The ability of statins to directly modify intracellular redox state in the human vascular endothelium [9, 10] leads to suppression of redox-sensitive transcriptional pathways (such as nuclear factor kappa B (NF-κB) and activator protein 1 (AP-1) in endothelial cells), that regulate the expression of multiple proinflammatory genes. This is an additional key mechanism by which statins exert their anti-inflammatory effect in vascular endothelial cells [54]. 

### Effects on Vascular Smooth Muscle Cells

Both intracellular redox state and pro-inflammatory stimuli activate NF-κB pathway in vascular smooth muscle cells (VSMCs) in a GGPP/FPP-dependent fashion [55]. In cultured VSMCs atorvastatin inhibits NF-κB activation by tumor necrosis factor-alpha (TNF-alpha) or angiotensin II (AngII) by restoring cytoplasmic levels of the NF-κB inhibitor IκB [54]. In rat aortic smooth muscle cells, atorvastatin negates thrombin-mediated increase in pro-inflammatory cytokine synthesis by inhibiting the membrane translocation of RhoA [56]. Thus statins reduce pro-inflammatory cytokines and chemokines release from VSMCs, including down-regulation of MMP-9 activity and expression that is responsible for extracellular matrix remodeling [57]. Statins also inhibit important intracellular pathways activated by Ang II, such as Rho-kinase and MAPK pathways, suppressing connective tissue growth factor expression in VSMCs, a mediator of vascular fibrosis [58]. Moreover statins inhibit proliferation [59] and migration of VSMCs [60] in a dose dependent fashion, thus suppressing intimal thickening in an animal model of vascular injury [60]. Statins effects on inhibition of VSMC proliferation are mediated by GGPP-reversible inhibition of Rho-kinase and DNA synthesis in VSMC [59]. Inhibition of extracellular signal-regulated kinases 1/2 activation by statins also blunts mitogenic pathways in VSMCs [61]. 

### Composite Effects on Vascular Redox and Vasomotor Function

Vascular redox state, as determined by the balance of ROS generation by pro-oxidant sources and elimination by the antioxidant defense systems, is pivotal for global vascular homeostasis and atherosclerosis development. Suppression of ROS production by statins and alteration of vascular redox state is considered a pivotal mechanism responsible for the anti-inflammatory effects of statins on the vascular wall. Gene expression and activity of cellular antioxidant enzymes, such as catalase, superoxide dismutase and thioredoxin, has been shown to be up-regulated by statins [62-64]. Heme oxygenase-1, an important vascular anti-oxidant defense system, is also up-regulated *in vitro *in HUVECs and aortic endothelial cells after atorvastatin treatment [65]. 

Further to the enhancement of vascular antioxidant defenses, statins attenuate Ang II pro-oxidant effects by down-regulating AT_1_R expression, while a direct scavenging effect on O_2_^- ^has also been reported [29]. Moreover, statins reduce NADPH-oxidase activity which is a major ROS source in the vascular wall. Preoperative treatment with atorvastatin in patients undergoing CABG rapidly improves vein grafts redox state by suppressing vascular NADPH oxidase activity both in endothelium and in VSMCs [10]. This is mediated by a mevalonate-reversible inhibition of isoprenoids formation and membrane translocation of the small GTP protein Rac1 [10]. This effect on NADPH oxidase derived ROS lowers vascular oxidative stress and protects BH_4 _from oxidative catabolism, thus indirectly enhancing eNOS coupling. The mechanisms by which statins improve NO bioavailability are presented in Fig. (**1**). Improvement in vascular NO bioavailability in human vessels by statins has been demonstrated *in vivo,* by improved endothelium-dependent vasodilatation. Statins administration in patients with hypercholesterolemia [66], diabetes mellitus [67], stable coronary artery disease [11], acute coronary syndromes [12] or heart failure [68] has been consistently shown to improve endothelial function. 

### Effects on Inflammatory Cells

In an *in vitro* model of lipopolysaccharide-induced inflammation, statins significantly decreased VSMC – monocytes interaction and their synergistic production of pro-inflammatory cytokines such as interleukin (IL)-6 [69]. Despite the lack of effects on animals, statins increased the number of CD4(+)CD25(+) regulatory T cells in humans which have a beneficial role in atherosclerosis-related inflammatory response [70]. In isolated T cells from healthy subjects, lovastatin inhibited cytokine production of IL-2, IL-4, and interferon (IFN)-γ from activated cells via down-regulation of both AP-1 and NF-κB in a dose dependent manner [71]. In lipopolysaccharide-stimulated B lymphocytes lovastatin inhibited the proliferation and differentiation of B cells and induced cells’ apoptosis [72]. 

In animal models of allograft atherosclerosis, statins reduce inflammatory cell infiltration in the arterial wall by reducing important chemokine expression such as Regulated upon Activation, Normal T-cell Expressed, and Secreted (RANTES) and Monocyte Chemo-attractant Protein-1 (MCP-1) expression, independently of intracellular cholesterol metabolism [73]. Simvastatin reduces the expression of pro-inflammatory cytokines such as IL-6, IL-8, and MCP-1 in peripheral blood mononuclear cells from patients with hypercholesterolemia, both *in vitro* and *in vivo *[74]. Macrophage inflammatory protein-1alpha has a well-established role in inflammatory response of atherogenesis and its expression is suppressed by atorvastatin in phorbol myristate acetate–activated THP-1 monocytes [75]. 

### Suppression of Platelet Activation

Platelet activation is closely linked to vascular inflammation and it is an essential element of atherogenesis. Activated platelets enhance chemotaxis of inflammatory cells and vascular wall inflammation via release of pro-inflammatory mediators. CD40/CD40 ligand plays a pivotal role in atherothrombosis by regulating endothelium – platelet interactions [76]. Incubation of activated platelets with atorvastatin leads to decreased platelet-induced cycloxygenase-2 expression in HUVECs via reduction of CD40 ligand surface expression [77]. In cell cultures pitavastatin attenuates platelet-derived growth factor, a promoter of VSMCs migration in atherogenesis [78]. Preservation of adenine nucleotide metabolism by modification of CD39/ATPDase expression of endothelial cells in a Rho-GTPase dependent pathway is another mechanism responsible for the reduced platelets-endothelium interactions induced by statins [79]. Statins also reduce platelet aggregation by altering intra-platelet redox state. Treatment of hypercholesterolemic patients with fluvastatin reduces ADP-induced platelet aggregation, increases platelet-derived NO synthesis and elevates intra-platelet glutathione and reduced to oxidized glutathione ratio in a GGPP-inhibitable manner [80]. PPAR-α and PPAR-γ stimulation [30] and reduced expression of CD36 and lectin-like oxidized-LDL receptor-1 in platelets surface [81] have been suggested as additional mechanisms by which statins reduce platelet activation. The acute, direct inhibitory effects of statins on platelet function have been also tested in an experimental porcine model, where intravenous lovastatin reduced the size of platelet-rich thrombus in the injured carotid artery by more than 50% [82]. 

### Plaque Stabilization 

Increased lipid content of the atheromatous plaque not only causes mechanical instability but also favors oxidative modification of lipids to oxidized LDL (ox-LDL) that further augment local inflammation and vascular oxidative stress [83]. It is widely accepted that statin-mediated lipid lowering stabilizes atheromatous plaques by mechanisms largely independent of LDL-lowering [83]. Immunohistochemistry studies of human carotid plaques have demonstrated that statins decrease CD68+ macrophages’ and CD3+ lymphocytes’ infiltration, increase collagen content, reduce MMPs activity and increase tissue inhibitor of metalloproteinase 1 (TIMP-1) expression [13, 84]. Modulation of MMPs activity has been suggested to be mediated by changes in the expression of small leucine-rich proteoglycans, such as decorin and biglycan, which might represent novel targets of statin treatment contributing to a stable plaque phenotype [85]. Concurrent suppression in the activity of inducible cyclooxygenase and prostaglandin E synthase also contribute to the composite anti-inflammatory and plaque stabilizing effect of statins [13]. Suppression of local plaque inflammation is reflected on reduced thermal heterogeneicity of atheromatous plaques of coronary patients under statin treatment [86]. 

### Suppression of Myocardial Inflammation

HMG-CoA reductase inhibitors modulate transcriptional factors activities in myocardial tissue. In a murine model of viral myocarditis, atorvastatin decreased myocardial TNF-α and IFN-γ and increased connexins expression, resulting in improved survival [87]. Other experimental studies suggest that pre-treatment with simvastatin significantly reduced systemic and myocardial levels of pro-inflammatory cytokines post cardiopulmonary bypass by stimulating PPAR-γ receptors and inhibiting NF-κB expression in myocardial tissue [31]. Further to the reduction of pro-inflammatory cytokines expression, statins up-regulate the expression of anti-inflammatory cytokine IL-10 and improve the balance between TNF-α / IL-10 post-myocardial infarction (MI) in rats. These effects ameliorate early left ventricle remodeling post-MI and improve left ventricular function [88]. The anti-inflammatory effects of statins on cardiac tissue prevent cardiac fibrosis and hypertrophy in animal models [89]. Failing human myocardium is characterized by increased generation of ROS predominantly due to increased NADPH oxidase and Rac1-GTPase activity and can be efficiently targeted with statins [90]. Statins significantly decreased p53-mediated apoptosis of cardiomyocytes in a murine model of sepsis [91]. Rosuvastatin prevented cardiomyocyte apoptosis in the peri-infarcted zone of pig hearts by activating RISK kinases and thus reduced the extent of damaged myocardium and improved heart function [92]. In another murine model, rosuvastatin suppressed local inflammation and attenuated the decrease of sarco/endoplasmic reticulum Ca^2^+-ATPase in the peri-infarction zone, preventing left ventricular remodeling and dysfunction [93]. Modulation of cytokine production from circulating T-helper (Th)-1 lymphocytes might also be another mechanism responsible for improvement of cardiac function post acute MI with statins treatment [94]. 

Atrial fibrillation is characterized by increased Rac1-GTPase activity. In human atrial tissues, atorvastatin inhibits Rac1-mediated activation of Nox2 NADPH-oxidase, lowers atrial superoxide generation and reduces the risk of atrial fibrillation post-CABG [95]. In contrast, statins do not affect atrial ROS generation from uncoupled NOS isoforms and mitochondrial oxidases [95]. Further to the suppressive effects of statins on myocardial inflammation and ROS generation, beneficial direct effects on atrial ion currents induce a favorable atrial electrical remodeling [96]. 

### Anti-Inflammatory Effects in Other Tissues

Rac1 inhibition by statins in endothelial cells induces the expression of a wide range of genes implicated in neurovascular protection and neural cell survival through increased release of neurotrophic factors [97]. Rosuvastatin up-regulates vascular eNOS which results in smaller cerebral infarction areas following middle cerebral artery occlusion in mice [98]. Importantly, by reducing the expression of major histocompatibility complex class II and the activation of antigen-specific T cells, atorvastatin reduces central nervous system inflammation and has beneficial effects on a murine model of experimental autoimmune encephalomyelitis [99]. Experimental evidence suggests that statins may be effective in reducing synovial tissue inflammation in autoimmune arthritis as they suppress Th1-related immune responses and IFN-γ release from mononuclear cells [100]. In patients with rheumatoid arthritis (RA), NF-κB activation in synoviocytes is RhoA kinase dependent. Simvastatin inhibits TNF-α-mediated activation of NF-κB in rheumatoid synoviocytes *in vitro* and reduces pro-inflammatory cytokine expression [101]. A RhoA-independent inhibition of MCP-1 expression in synoviocytes by statins has also been reported [102]. 

## ANTI-INFLAMMATORY EFFECTS OF STATINS AND CLINICAL OUTCOME

### Non-lipid lowering effects of statins and cardiovascular risk reduction: early evidence from clinical trials

The benefits from lipid-lowering with statins in primary [2, 3] and secondary [4, 5] prevention of CVD have been established in large randomized clinical studies. A summary of all the important randomized clinical trials on statins [2-4, 14, 15, 21, 26-28, 103-112] in primary or secondary prevention of CVD is provided in Table **1**. Some of these landmark clinical trials yielded the early clinical evidence for the beneficial effects of statins independent of their lipid lowering properties. In the LIPID (Long-Term Intervention with Pravastatin in Ischaemic Disease) trial, cardiovascular risk reduction was significant throughout the continuum of LDL cholesterol levels, even in patients with LDL cholesterol <100mg/dL [4]. In addition the Air Force/Texas Coronary Atherosclerosis Prevention Study (AFCAPS/TexCAPS) trial demonstrated that lovastatin reduced cardiovascular events in patients with low LDL cholesterol and high CRP levels, while it conferred no clinical benefit in patients with both low LDL cholesterol and low CRP levels [2]. These findings suggested that patients with increased low-grade systemic inflammation (as determined by high sensitivity CRP levels) could constitute an additional group that could benefit from statin treatment, irrespectively of baseline LDL cholesterol levels.

Moreover, in a pooled analysis of 3 of these large clinical studies [3-5], further to the reduction of CHD risk, pravastatin also reduced the risk of stroke [22]. This had significant clinical implications because the risk of stroke had been poorly correlated with cholesterol levels in large epidemiological studies [113]. In the SPARCL (Stroke Prevention by Aggressive Reduction in Cholesterol Levels) trial, high dose atorvastatin treatment (80mg/day) in patients with history of stroke or transient ischemic attack but without known CHD improved cardiovascular outcomes. After a mean of 4.9 years atorvastatin reduced ischemic stroke risk by 33% and risk of major coronary events by 37% [21].

The MIRACL (Myocardial Ischemia Reduction with Aggressive Cholesterol Lowering) trial yielded additional evidence on the anti-inflammatory effects of statins in high risk subjects [14]. High dose atorvastatin treatment (80mg/day) for 16 weeks in patients with unstable angina or non-Q-wave MI 24 to 96 hours post hospital admission, significantly reduced ischemic events, particularly symptomatic ischemia [14]. This population had low baseline LDL cholesterol levels (124mg/dL) that were significantly reduced at 16 weeks (to 72mg/dL). The baseline levels of inflammatory markers such as CRP and serum amyloid alpha independently predicted the risk of stroke [108], and atorvastatin reduced the levels of these biomarkers [114]. Timing and dosage selection of statins in acute coronary syndromes (ACS) seem to influence treatment efficacy as early administration of high dose simvastatin confers more potent anti-inflammatory effects during the first week after an acute MI event [115]. Additional loading with high-dose atorvastatin before percutaneous coronary intervention (PCI) further reduces periprocedular myocardial injury and 30-day cardiovascular morbidity in patients who are already on chronic statin treatment [116].

These important findings gave strong evidence that statin treatment could be extended to subjects without hypercholesterolemia for CHD prevention based on their anti-inflammatory, plaque stabilizing and overall vasoprotective effects. Statins could also be used outside the strict context of CHD prevention, as in the primary or secondary prevention of stroke. Although these studies provided some evidence on the clinical benefits by statins in patients with low-grade vascular inflammation, they were not designed to specifically address this issue. The most convincing evidence for improvement of clinical outcome by statins due to their anti-inflammatory effect was provided by the JUPITER trial and will be discussed further [26]. 

### Suppression of Inflammation by Statins and Cardiovascular Risk: The JUPITER Trial and Beyond

JUPITER [26] was the first trial specifically designed to address whether the anti-inflammatory effects of statins could alter clinical outcome in healthy, normolipidemic subjects with elevated CRP levels. In total, 17,802 apparently healthy men and women with low LDL cholesterol levels (<130mg/dL) and increased CRP levels (>2.0mg/L) were randomized to rosuvastatin (20mg/day) or placebo [26]. The trial was stopped prematurely after a median follow-up of 1.9 years due to an interim analysis suggesting significant clinical benefits in the rosuvastatin group. Rosuvastatin reduced LDL cholesterol levels by 50% and hs-CPR levels by 37% and conferred a risk reduction of 0.56 (95% confidence interval [CI], 0.46 to 0.69; P<0.00001) for the combined primary end point. The study demonstrated that even in low or intermediate risk patients according to Framingham risk score, without hypercholesterolemia, suppression of background inflammation by statins prevents CVD development and improves clinical outcome [26]. JUPITER has received criticism for not having included also a group with low CRP levels and for its short follow-up period [117]. Nevertheless JUPITER remains a landmark literature since it highlighted that suppression of inflammation by statins may be considered as an additional treatment target for prevention of CVD, in conjunction to the other traditional risk factors.

Findings of JUPITER urged United States Food and Drug Administration to approve rosuvastatin administration in subjects with elevated CRP levels and at least one additional risk factor for CHD. On the other hand European Health Authorities approved rosuvastatin use for primary prevention of CVD in high risk patients as determined by a Framingham risk score>20% or systematic coronary risk evaluation (SCORE) risk ≥5% [118]. In a recent *post hoc* analysis of JUPITER, Koenig and Ridker [118] examined the effects of rosuvastatin administration in high cardiovascular risk patients of JUPITER according to European guidelines (i.e. those with a Framingham risk score>20% or SCORE risk ≥5%). In that analysis it was shown that rosuvastatin significantly decreased cardiovascular risk overall [118]. In patients with a Framingham risk >20% risk reduction was HR=0.50 (95% CI: 0.27–0.93, P = 0.028), while in patients with SCORE risk ≥5% risk reduction was HR=0.57 (95% CI: 0.43–0.78, P = 0.0003) [118]. 

A recent analysis of 20,536 participants of the Heart Protection Study also demonstrated that simvastatin administration for 5 years significantly reduced risk of major vascular events independently of baseline serum CRP and LDL levels [119]. Simvastatin treatment conferred significant cardiovascular risk reduction even in subjects with CRP levels <1.25mg/L [119]. 

### Reducing Inflammation in Heart Failure and Chronic Kidney Disease: Lessons from GISSI-HF, CORONA and AURORA

Findings from the JUPITER suggest that other groups of patients with high inflammation, such as heart failure and chronic kidney disease (CKD) patients may also benefit from statins treatment. However, there has been conflicting evidence reported in literature. 

Statins have potent anti-inflammatory effects on myocardium as well as beneficial effects in heart failure patients such as improvement of symptoms severity, B natriuretic peptide (BNP) levels and endothelial function [68, 120]. Nevertheless the effect of statins on clinical prognosis of heart failure patients remains equivocal. The first important randomized clinical trial on statins in heart failure patients was The Controlled Rosuvastatin Multinational Trial in Heart Failure (CORONA) [28]. In total, 5,091 patients with ischemic systolic heart failure (NYHA class II-IV) were randomized to receive rosuvastatin (10mg/day) or placebo and were followed-up prospectively for a median of 2.7 years. Despite significantly decreasing LDL cholesterol and CRP levels and reducing cardiovascular hospitalizations, rosuvastatin failed to reduce the primary outcome (death from cardiovascular causes, nonfatal myocardial infarction, or nonfatal stroke) or the number of deaths from any other cause [28]. The GISSI-HF (Gruppo Italiano per lo Studio della Sopravvivenza nell'Insufficienza Cardiaca) study was another placebo-controlled randomized clinical trial in 4,574 heart failure patients that yielded similar results. In this trial, which also included patients with non-ischemic and/or diastolic heart failure (NYHA class II-IV), rosuvastatin (10mg/day) failed to improve cardiovascular outcomes after a median of 3.9 years [15]. 

Patients with CKD are at increased risk for developing CVD. There is evidence that the anti-inflammatory and pleiotropic effects of statins may preserve renal function and reduce proteinuria in CKD [121]. Meta-analysis of the three large clinical trials from the Pravastatin Pooling Project suggested that pravastatin reduced cardiovascular events in people with (or at risk for) CVD and concomitant moderate CKD [121]. Similarly an analysis of JUPITER suggested that rosuvastatin significantly reduced first cardiovascular events and all-cause mortality in subjects with moderate CKD [122]. Nevertheless, these positive findings have been opposed by the findings of the AURORA trial in hemodialysis patients [27]. In this trial 2,776 hemodialysis patients were randomized to rosuvastatin (10mg/day) or placebo and were followed-up for a median of 3.8 years. Rosuvastatin had no effect on the composite primary endpoint of death from cardiovascular causes, nonfatal MI, or nonfatal stroke [27]. 

Despite the original assumption that statins would improve patients’ prognosis with CKD or heart failure, the CORONA, GISSI-HF and AURORA trials yielded unexpected negative results. Notably, statins administration could also have adverse effects in heart failure patients given the association between low cholesterol levels and poor outcome in advanced heart failure [123]. Other considerations against statins use in heart failure include their adverse effects on coenzyme Q10 (ubiquinone) and selenoproteins that are important for myocardium biology. Nevertheless the CORONA study failed to detect a group of patients that was adversely affected by rosuvastatin treatment [124]. A *post hoc* analysis of CORONA suggested that rosuvastatin was beneficial in the group of patients with baseline CRP levels ≥2.0mg/L. No such benefit was observed in the group of patients with CRP<2.0mg/L [125]. Moreover, in a subgroup analysis stratified by BNP levels, patients in the lowest tertile of NT-proBNP levels (<868pg/mL) had the best prognosis and rosuvastatin significantly reduced primary endpoint in these patients [126]. These findings indicate that there is a group of heart failure patients that may indeed benefit from statins treatment. An explanation for these findings is that statins might exert beneficial effects only in the early stages of CVD continuum, as in low risk subjects without CVD and early stage heart failure patients, but not in the case of end-stage heart failure or in hemodialysis patients [126]. Besides it should be noted that both in heart failure and hemodialysis patients, rosuvastatin was safe and well tolerated, suggesting that there is no reason to withdraw statins administration in these settings. Even though no clear benefit in outcome should be expected, reduction in cardiovascular hospitalization [28] or reduced risk of atrial fibrillation development [25] might be secondary gains with statin use. CRP and NT-proBNP levels are particularly helpful in detecting those heart failure patients more likely to benefit from statin treatment. However the results should be interpreted with caution as they were derived from *post hoc* analyses. It should be also noted that the existing trials have been limited to rosuvastatin administration and investigation of other statins is lacking. A former trial in CKD patients [127] also failed to demonstrate any effects of atorvastatin on cardiovascular outcomes. However, a recent large trial in patients with advanced CKD (3,023 dialysis vs. 6,247 non-dialysis patients) without known CHD demonstrated that combined simvastatin (20mg/day) and ezetimibe (10mg/day) administration for a median of 4.9 years reduced the incidence of major atherosclerotic events (rate ratio 0.83, [95% CI 0.74-0.94]; p=0.0021) [128]. Despite these positive findings, the concomitant use of ezetimibe makes the interpretation of these results particularly challenging. Additional clinical trials are required to elucidate this issue along with cost-effectiveness aspects of statin use in these groups of high cardiovascular risk patients. 

## REBOUND INFLAMMATION AFTER STATIN WITHDRAWAL?

The largely negative findings of the recent randomized clinical trials with statins in heart failure and CKD have revamped the interest in the phenomenon of statin withdrawal. Experimental evidence suggests that statin withdrawal rapidly abrogates the beneficial effects of statins and induces rebound inflammation. In mice, statin withdrawal results in a negative feedback up-regulation of Rho gene transcription, increasing Rho activity and suppressing endothelial NO production [129]. In hypercholesterolemic patients, atorvastatin withdrawal rapidly increases pro-inflammatory and pro-thrombotic pathways [130], while simvastatin withdrawal during an acute MI event has been associated with a rebound increase in CRP levels [131]. Retrospective data from clinical registries have suggested that statins discontinuation in non-ST segment elevation MI patients is associated with worse clinical outcomes [19]. Even though this rebound inflammatory response after statins withdrawal does not alter cardiovascular risk in stable coronary patients [132], it may induce plaque instability and subsequent adverse events in ACS. Statin withdrawal in the perioperative period of major vascular surgery has also been associated with an increased risk for perioperative adverse cardiac events [133]. Ethical issues thus hinder the conduction of randomized clinical trials on statins withdrawal. The sole prospective, randomized controlled trial on statins withdrawal has been conducted in patients with a hemispheric ischemic stroke [134]. The study concluded that stopping statins for 3 days is associated with increased risk of death or dependency, greater neurological deterioration and a larger infarct volume [134]. Despite the lack of evidence from randomized clinical studies it seems rational to avoid statins discontinuation in high cardiovascular risk patients, unless strong contraindications exist. 

## STATINS AS ANTI-INFLAMMATORY AGENTS IN OTHER CLINICAL CONDITIONS

In the light of ample experimental evidence statins seem an attractive option for treatment of patients with autoimmune or inflammatory diseases. Indeed clinical evidence suggests that statins could be beneficial in a number of pathological disorders such as in osteoporosis and osteoporotic bone fractures, Alzheimer’s disease, Parkinson’s disease, multiple sclerosis, organ transplantation, rheumatic diseases, allergic asthma, sepsis and others [135]. Statins administration post lung transplantation has been associated with improved survival and improved graft function [136]. In acute pericarditis addition of rosuvastatin to indomethacin treatment results in faster reductions in CRP levels, pericardial effusion and ST segment normalization, although it does not alter hospitalization length [137]. A small clinical trial has reported that simvastatin may stabilize neurologically patients with relapsing-remitting multiple sclerosis that are poor responders to interferon beta-1a therapy [138]. However a recent clinical trial found no benefit of simvastatin as add-on therapy to interferon beta-1a in multiple sclerosis. Studies suggest that similarities exist between osteoporosis and atherosclerosis development [139]. Calcification is a common feature of atherosclerotic plaques, and osteoporosis is associated with both atherosclerosis and vascular calcification [140]. Evidence suggests that statins could favorably affect bone formation and prevent osteoporotic bone fractures, however this has not been investigated yet in large prospective clinical studies [140]. Before certain conclusions can be drawn for each one of these disease states, evidence from additional clinical trials is required [141]. 

### Statins in Rheumatic Diseases

Given their anti-inflammatory and immunomodulatory properties statins have drawn much attention as promising agents in the treatment of rheumatic diseases [142]. In animal models statins delay the onset of collagen-induced arthritis by reducing synovial tissue inflammation [143]. In humans, epidemiological data suggest that statins use is associated with lower risk of RA development [144]. A cohort study on 211,627 subjects that initiated statins treatment suggested that compared to non-compliant users, compliance with statin treatment was associated with significantly lower risk for RA development (HR=0.58 95%CI, 0.52-0.65) [144]. Further to lowering risk of RA development, clinical evidence suggests that statins also favorably modulate disease activity. A large observation study in RA patients demonstrated that the use of statins is associated with reduced CRP levels and lower disease activity [145]. The randomized clinical Trial of Atorvastatin on Rheumatoid Arthritis (TARA) also reported beneficial effects of atorvastatin on disease activity and systemic inflammation [146].

Further to possible benefits on disease activity, statin therapy in patients with rheumatic diseases is justified as a means to lower vascular risk. Patients with inflammatory arthritis are at increased risk for development of atherosclerotic abnormalities [141]. This increased vascular risk in patients with inflammatory arthritis partly stems from synovial tissue inflammation that leads to a systemic inflammatory response and vascular endothelium inflammation [147]. It has been estimated that increased CVD risk of RA patients is close to that of moderate hypercholesterolemia or arterial hypertension [147]; therefore presence of RA cannot be considered as a CHD risk equivalent [147]. JUPITER trial excluded individuals with diagnosed rheumatic diseases and other chronic inflammatory conditions and therefore direct extrapolations to rheumatic disease patients cannot be made. Nevertheless since these patients are characterized by increased systemic inflammation, it has been suggested that RA can be considered as an additional CVD risk factor, particularly in those patients with CRP levels >3mg/L [147]. Besides evidence suggests that when CVD risk is evaluated in RA patients, depending on the model used for cardiovascular risk stratification 2% to 26% of RA patients without CVD are at sufficiently high risk to require statin therapy [148]. Nevertheless most of them remain untreated [148]. A *post hoc* analysis of IDEAL trial suggested that, despite having lower baseline cholesterol levels, MI patients with RA had similar cardiovascular event rates to those without RA [149]. In these RA patients, atorvastatin and simvastatin were safe and efficient agents, conferring lipid-lowering effects comparable to that induced in patients without RA [149]. Further to their strong lipid lowering effects, statins also improve endothelial function in patients with RA [150]. Another study however suggested that fibrates, but not statins, significantly reduce cholesterol levels in RA patients [151]. In patients with systemic lupus erythematosus pravastatin significantly reduces cholesterol levels (even though less effectively in patients under glucocorticoids’ treatment or with higher body mass index) but induces no effects on CRP levels [152]. These findings suggest that the beneficial effects of statins may differ among the different compounds of statins used or according to the type of the underlying rheumatic disease. 

Nevertheless, given the well-established effect of statins on vascular risk, a thorough CVD risk evaluation should be undertaken in all patients with rheumatic disease, and when justified, statins treatment could be considered as an adjunct therapy to lifestyle interventions [147]. Further clinical studies are required to evaluate better CVD risk stratification models in patients with different types of rheumatic disease and examine the possible beneficial effects of statins on the clinical outcome of these patients [141]. 

## CONCLUSIONS

The anti-inflammatory effects of statins on vascular wall are now widely accepted. By inhibiting intracellular isoprenoids formation, statins suppress vascular and myocardial inflammation, favorably modulate vascular and myocardial redox state and improve nitric oxide bioavailability. Randomized clinical trials have demonstrated that further to their lipid lowering properties, statins also reduce cardiovascular risk by exerting anti-inflammatory effects. However, the extent to which the beneficial effects of statins on clinical outcome are lipid-lowering independent remains unclear. Early initiation of statin treatment in normolipidemic subjects without cardiovascular disease and elevated C-reactive protein levels has a significant favorable impact on cardiovascular risk. The neutral effect of rosuvastatin in heart failure and chronic kidney disease patients suggests that careful selection of the treated population should be made, to avoid unnecessary treatments. Further research is required in order to clarify the impact of statin treatment in these clinical conditions and to establish their role in other inflammatory diseases. 

## Figures and Tables

**Fig. (1):**
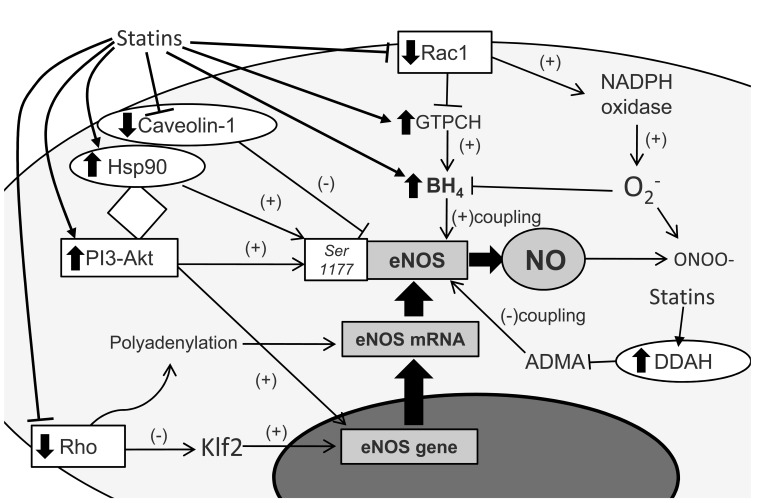
Statins and nitric oxide bioavailability. Statins favorably affect eNOS gene expression, eNOS mRNA and protein levels and eNOS coupling. By inhibiting intracellular isoprenoids formation, statins reduce the activation of the small GTPase Rho protein, resulting in increased eNOS gene expression via Klf2 and eNOS mRNA stabilization by its polyadenylation. eNOS gene expression is also increased via stimulation of the PI3-Akt pathway by statins. PI3-Akt pathway may be also enhanced by Hsp90 upregulation by statins. Both PI3-Akt and Hsp90 induce eNOS protein phosphorylation at *Ser1177* that increases eNOS activity. Caveolin-1 regulates subcellular localization of eNOS and inactivates the enzyme. Statins reduce expression of caveolin-1 and therefore increase cytosolic abundance of eNOS. Intracellular HMG-CoA reductase inhibition by statins increases *GCH1* mRNA expression, the gene that encodes GTPCH, the rate limiting enzyme in BH_4_ biosynthesis that is critical for eNOS coupling. Statins also indirectly improve eNOS coupling by lowering vascular O_2_^-^ generation. Rac1 inactivation by statins inhibits NADPH-oxidase activity and NAPDH-oxidase derived O_2_^-^ while a direct scavenging of O_2_^-^ by statins has also been reported. O_2_^-^ reduces NO bioavailability by reacting with NO to form ONOO^-^ the latter being mainly responsible for BH_4_ oxidation. Finally, increased DDAH by statins -the enzyme responsible for ADMA catabolism- results in lower ADMA levels and improved eNOS coupling. ADMA: asymmetric dimethylarginine, BH4: tetrahydrobiopterin, DDAH: dimethylarginine dimethylaminohydrolase, eNOS: endothelial nitric oxide synthase, GTPCH: GTP-cyclohydrolase I, HMG-CoA: 3-hydroxy-3-methylglutaryl coenzyme A, Hsp90: Heat shock protein 90, Klf2: Kruppel-like factor 2, NO: nitric oxide, O_2_^-^: superoxides, ONOO^-^: peroxynitrite, (-): inhibits/suppresses, (+): increases.

**Table 1.** Important Randomized Clinical Trials on Statins and Clinical Outcomes in the Context of Cardiovascular Disease

Study	Population	Treatment	Outcome
WOSCOPS [3]	6,595 men without CHD	Pravastatin *vs*. placebo	Pravastatin reduced coronary events by 31% (95%CI, 17-43%), and coronary mortality by 32% (95%CI, 3-53%)
AFCAPS / Tex-CAPS [2]	6,605 subjects without CHD	Lovastatin *vs*. placebo	Lovastatin reduced incidence of first acute major coronary events (RR=0.63; 95%CI, 0.50-0.79)
SPARCL [21]	4,731 TIA/stroke patients	Atorvastatin *vs*. placebo	Atorvastatin reduced stroke risk (HR=0.84; 95%CI, 0.71-0.99) and MACE risk (HR=0.80; 95%CI, 0.69-0.92)
JUPITER [26]	17,802 subjects (LDL<130mg/d, CRP>2.0mg/L	Rosuvastatin *vs*. placebo	Rosuvastatin reduced risk for MACEs (HR=0.56; 95% CI, 0.46-0.69)
ALLHAT-LLT [104]	10,355 hypercholesterolemic, hypertensive patients	Pravastatin *vs*. usual care	No effect of pravastatin on either all cause mortality or CHD
ASCOT-LLA [112]	1,9342 hypertensive patients	Atorvastatin *vs*. placebo	Atorvastatin reduced risk for primary events (HR=0.64; 95%CI, 0.50-0.83)
4S [103]	4,444 CHD patients	Simvastatin *vs*. placebo	Simvastatin reduced risk for death (RR=0.70; 95% CI 0.58-0.85) and for coronary death (RR=0.58; 95% CI, 0.46-0.73)
LIPID [4]	9,014 CHD patients	Pravastatin *vs*. placebo	Pravastatin reduced coronary mortality risk by 24% (95%CI, 12-35%)
HPS [105]	20,536 patients with CHD, occlusive arterial disease, or diabetes	Simvastatin *vs*. placebo	Simvastatin reduced all cause and coronary mortality, and vascular events by 24% (95%CI 19-28%)
CARE [5]	4,159 MI patients	Pravastatin *vs*. placebo	Pravastatin reduced risk for nonfatal AMI by 24% (95%CI, 9-36%)
TNT [109]	10,001 CHD patients	Atorvastatin (high *vs*. low dose)	Reduced risk for MACEs with high dose treatment (HR=0.78; 95%CI, 0.69-0.89)
LIPS [110]	1,677 CHD patients	Fluvastatin *vs*. placebo	Fluvastatin reduced risk for MACEs (RR=0.78; 95%CI, 0.64-0.95)
FLARE [111]	1,054 patients undergoing PTCA	Fluvastatin *vs*. placebo	Significantly lower incidence of total death and AMI with fluvastatin
A to Z trial [107]	4,497 ACS patients	Simvastatin *vs*. placebo	Favorable trend toward reduction of MACEs with simvastatin
MIRACL [14]	3,086 UA or AMI patients	Atorvastatin *vs*. placebo	Reduced risk for recurrent ischemic events with atorvastatin
PROVE IT-TIMI 22 [106]	4,162 ACS patients	Atorvastatin *vs*. Pravastatin	Atorvastatin reduced risk for death or CV events by 16 % (95%CI, 5-26%).
CORONA [28]	5,011 HF patients	Rosuvastatin *vs*. placebo	No difference in coronary events or death. Fewer hospitalizations with rosuvastatin
GISSI-HF [15]	4,574 HF patients	Rosuvastatin *vs*. placebo	No effect on CV outcome
AURORA [27]	2,776 hemodialysis patients	Rosuvastatin *vs*. placebo	No effect on CV outcome
4D [127]	1,255 diabetics on hemodialysis	Atorvastatin *vs*. placebo	No effect on CV outcome
SHARP [128]	9,270 CKD patients	Simvastatin+ezetimibe * vs*. placebo	Reduced risk of major vascular events (RR=0.83, 95% CI 0.74-0.94) with simvastatin+ezetimibe

ACS: Acute coronary syndrome; AMI: acute myocardial infarction, CHD: coronary heart disease, CKD: chronic kidney disease, CRP: C-reactive protein, CV: cardiovascular, HR: hazard ratio, LDL: low density lipoprotein, MACE: major adverse cardiac events, PTCA: percutaneous transluminal coronary angioplasty, RR: relative risk, TIA: transient ischemic attack, UA: unstable angina
